# Feline Panleucopenia Virus NS2 Suppresses the Host IFN-β Induction by Disrupting the Interaction between TBK1 and STING

**DOI:** 10.3390/v9010023

**Published:** 2017-01-23

**Authors:** Hongtao Kang, Dafei Liu, Jin Tian, Xiaoliang Hu, Xiaozhan Zhang, Hang Yin, Hongxia Wu, Chunguo Liu, Dongchun Guo, Zhijie Li, Qian Jiang, Jiasen Liu, Liandong Qu

**Affiliations:** 1 Division of Zoonosis of Natural Foci, State Key Laboratory of Veterinary Biotechnology, Harbin Veterinary Research Institute, Chinese Academy of Agricultural Sciences, 678 Haping road, Xiangfang District, Harbin 150000, China; kang1989462@sina.com (H.K.); hattman324@163.com (D.L.); tj6049345@126.com (J.T.); Liang679@163.com (X.H.); xiaozhan063@163.com (X.Z.); whx450650@163.com (H.W.); liuchunguo@caas.cn (C.L.); gdongchun@126.com (D.G.); lizhijie.1982@126.com (Z.L.); jiangqian623@sina.com (Q.J.); neauljs@163.com (J.L.); 2 College of Veterinary Medicine, Northeast Agricultural University, Harbin 150000, China; yinhang_marine@126.com

**Keywords:** Feline panleucopenia virus (FPV), Nonstructural protein 2 (NS2), interferon β (IFN-β)

## Abstract

Feline panleucopenia virus (FPV) is a highly infectious pathogen that causes severe diseases in pets, economically important animals and wildlife in China. Although FPV was identified several years ago, little is known about how it overcomes the host innate immunity. In the present study, we demonstrated that infection with the FPV strain Philips-Roxane failed to activate the interferon β (IFN-β) pathway but could antagonize the induction of IFN stimulated by Sendai virus (SeV) in F81 cells. Subsequently, by screening FPV nonstructural and structural proteins, we found that only nonstructural protein 2 (NS2) significantly suppressed IFN expression. We demonstrated that the inhibition of SeV-induced IFN-β production by FPV NS2 depended on the obstruction of the IFN regulatory factor 3 (IRF3) signaling pathway. Further, we verified that NS2 was able to target the serine/threonine-protein kinase TBK1 and prevent it from being recruited by stimulator of interferon genes (STING) protein, which disrupted the phosphorylation of the downstream protein IRF3. Finally, we identified that the C-terminus plus the coiled coil domain are the key domains of NS2 that are required for inhibiting the IFN pathway. Our study has yielded strong evidence for the FPV mechanisms that counteract the host innate immunity.

## 1. Introduction

Feline panleukopenia is an acute, highly contagious, and fatal infectious disease. The causative pathogen, feline panleucopenia virus (FPV), is a small (18–25 nm) negative-sense single stranded DNA virus, which has the widest host range and highest pathogenicity of the viruses in the carnivore parvovirus subgroup. Many carnivore parvoviruses, including canine parvovirus and mink enteritis virus, evolved from FPV to adapt into new hosts. Its genome includes two major open reading frames that express the nonstructural (NS) proteins (NS1 and NS2) and capsid protein (VP), including VP1 and VP2 by alternative splicing. The viruses replicate using the host cell polymerases and other DNA replication machinery, which causes the virus to infect the rapidly dividing cells [1,2]. In 2014, in Zhengzhou, Henan Province, and in 2015 in Guangyuan, Sichuan Province of China, it was reported that giant pandas were infected with FPV. Furthermore, in recent years, FPV has been epidemic in pets, economically important animals, and wildlife in China. Clinically, the disease has a high rate of infection, atypical symptoms, long-term infectious carriers and a significantly higher prevalence of co-infection with feline calicivirus (FCV) and feline herpesvirus 1 (FHV-1), which may be due to an insufficient host immune response against FPV infection, suggesting that FPV has the ability to antagonize the host antiviral response.

The innate immune system is the first line of host defenses against viral infection, and the induction of interferon (IFN)-α/β is a crucial antiviral mechanism of the innate immune system, which plays an important role in the defense against invading viruses, the termination of early viral replication and the development of an adaptive immune response [3,4]. The initiation of IFN expression is triggered by pathogen-associated molecular patterns (PAMPs) through host pattern recognition receptors (PRRs) [5,6]. When a host is invaded by a virus, PRRs transmit signals to different downstream adaptor molecules such as mitochondrial antiviral-signaling protein (MAVS), TIR-domain-containing adapter-inducing interferon-β (TRIF), and MyD88 (myeloid differentiation primary response gene 88) to recruit IκB kinase (IKK)-related kinases. With the help of the IKK-related kinases, transcription factors, including IFN regulatory factors (IRFs), subunits of the nuclear factor (NF)-κB/Rel family, and ATF-2/c-Jun (AP-1), are activated by phosphorylation and translocate into the nucleus. Once in the nucleus, the transcription factors bind to their specific binding elements, termed positive regulatory domains (PRDs), within the IFN-β promoter region to initiate IFN transcription [7,8].

Due to the powerful antiviral effect of type I interferon, the virus must encode some components that inhibit the host interferon signaling pathway for survival during the co-evolution of the virus and host. In the present study, we found that FPV NS2 could inhibit Sendai virus (SeV) mediated IFN-β induction by disrupting the TBK1–STING interaction and identified that the C-terminus plus the coiled coil domain are the regulatory elements of NS2 that inhibit the IRF3 signaling pathway. To the best of our knowledge, there is no related research about the inhibition of type I interferon induction by FPV. Our work reveals a novel mechanism that explains how FPV NS2 efficiently inhibits host innate immunity.

## 2. Materials and Methods

### 2.1. Viruses and Cells

F81 cells were obtained from the Cell Resource Center of the Shanghai Institutes for Biological Science, Chinese Academy of Science, and maintained in Roswell Park Memorial Institute (RPMI) 1640 medium with 10% fetal bovine serum (Life Technologies, San Diego, CA, USA) at 37 °C with 5% CO_2_. The FPV strain Philips-Roxane was obtained from American Type Culture Collection (ATCC).

### 2.2. Plasmids

The plasmids pIFN-β-Luc, p3×PRDII-Luc, PRDIII/I-Luc, and p6×PRDIV-Luc contain a luciferase (Luc) expression cassette driven by the feline IFN-β promoter, three copies of the NF-κB binding region, three copies of the IRF3 binding region and six copies of the AP-1 binding region, respectively, which have been described previously [9]. The pRL-TK plasmid (Promega, Madison, WI, USA), a vector that encodes Renilla luciferase, was used as an internal control for the normalization of gene transfection efficiency. The p3×Flag-MAVS and p3×Flag-STING vectors encode the Flag-tagged feline MAVS and STING proteins, respectively [10,11]. The p3×Flag-VP1, p3×Flag-VP2, p3×Flag-NS1, and p3×Flag-NS2 plasmids express Flag-tagged FPV VP1, VP2, NS1, and NS2, respectively. The pMyc-TBK1 plasmid produces the Myc-tagged feline TBK1. The pEGFP-IRF3-5D plasmid expresses the feline IRF3 with an EGFP tag. The feline TBK1 sequence was amplified from the plasmid pMyc-TBK1 and inserted into the pDsRed2-C1 plasmid (pDsred-TBK1). The FPV NS2 sequence was amplified from the plasmid p3×Flag-NS2 and inserted into the pEGFP-C1 plasmid (pEGFP-NS2) and pcDNA™3.1/V5-Hi plasmid (pV5-NS2).

### 2.3. Transfections and Luciferase Reporter Assays

F81 cells were seeded in 48-well plates prior to transfection and then co-transfected with luciferase reporter plasmids, the internal control pRL-TK plasmid and various expression plasmids or an empty control plasmid. After 24 h of co-transfection, the cells were inoculated with SeV at an MOI of 10 as an interferon pathway activator. The cells were lysed with 65 μl of Passive Lysis buffer (Promega) 12 h after stimulation, and the supernatants were used to measure firefly and Renilla luciferase activities using the Dual Luciferase Assay System according to the manufacturer’s instructions (Promega). The data are presented as relative firefly luciferase activities normalized to Renilla luciferase activities (means ± SD) and are representative of three independent experiments.

### 2.4. Western Blotting

The cell lysates were separated using 12% SDS-PAGE and then transferred onto nitrocellulose membranes (Millipore, Bedford, MA, USA). The membranes were blocked with tris-buffered saline Tween 20 (TBST) containing 5% skim milk for 2 h at room temperature (RT) and then incubated with the indicated primary antibodies for 1 h at RT or overnight at 4 °C. After being washed with TBST, the membranes were incubated with an IRDye 800-conjugated secondary antibody (Rockland Immunochemicals, Limerick, PA, USA) for 1 h at RT. The membranes were washed three times in TBST and then visualized and analyzed with an Odyssey Infrared Imaging System (LI-COR Biosciences, Lincoln, NE, USA). The intensities of bands were analyzed with Image J1.49 software.

### 2.5. Co-Immunoprecipitation (Co-IP) Analysis

F81 cells were co-transfected with pMyc-TBK1 and p3×Flag-NS2 or with pV5-NS2, pMyc-TBK1, and p3×Flag-STING using Lipofectamine 2000 (Invitrogen, Grand Island, NY, USA). Thirty-six hours later, the cells were lysed in radioimmunoprecipitation assay (RIPA) lysis buffer (50 mM Tris–HCl, pH 7.4, 150 mM NaCl, 1% NP-40, 0.5% sodium deoxycholate, 0.1% SDS, 1 mM phenylmethylsulfonyl fluoride (PMSF)) and incubated on ice with shaking. Next, the cell lysates were centrifuged for 10 min at 12,000 g. The supernatants were incubated with equilibrated ANTI-FLAG M2 magnetic beads (Sigma-Aldrich, Saint Louis, MO, USA) for 1 h at RT with gentle mixing. After the binding step, the magnetic beads were collected by a magnetic separator, and the supernatant was removed. Then, the bead-protein complexes were washed with TBS buffer to remove all of the nonspecifically bound proteins. The proteins were eluted from the beads in 40 μL of 2×SDS-PAGE sample buffer and subjected to boiling for 10 min. The proteins were separated by SDS-PAGE and transferred to nitrocellulose membranes for western blot analyses. Each experiment was repeated at least three times.

### 2.6. Separation of Cytoplasmic and Nuclear Extracts

The cytoplasmic and nuclear extracts of F81 cells were separated using NE-PER^®^ Nuclear and Cytoplasmic Extraction Reagents according to the manufacturer’s instructions (Thermo Fisher Scientific, Rockford, IL, USA). Briefly, after being transfected with p3×Flag-NS2 and stimulated with SeV the F81 cells were harvested with trypsin-EDTA and then centrifuged at 500× g for 5 min. After centrifugation, the cell pellets were washed with phosphate buffered saline (PBS), and the supernatant was discarded. Next, the cell pellets were homogenized in Cytoplasmic Extraction Reagent I (CER I) (Thermo Fisher Scientific) and vortexed to fully suspend the cell pellet. The cell pellets were then incubated on ice. Cytoplasmic Extraction Reagent II (CER II) was added to the cell pellet. After vortexing and centrifugation, the supernatants (cytoplasmic extract) were transferred to a clean tube. The insoluble (pellet) fraction was suspended in Nuclear Extraction Reagent (NER). After vortexing and centrifugation, the supernatants (nuclear extract) were transferred to a clean tube. The cytoplasmic and nuclear extracts were analyzed by western blotting.

### 2.7. Subcellular Localization

To further investigate the interaction between the feline TBK1 protein and FPV NS2, F81 cells were plated in Nunc^®^ glass-bottom dishes (Sigma-Aldrich) and co-transfected with the pDsred-TBK1 plasmid with red fluorescence and pEGFP-NS2 plasmid with green fluorescence. Twenty-four hours after transfection, the cells were washed with PBS, fixed with 4% paraformaldehyde at 4 °C for 30 min and then permeabilized with 0.2% Triton X-100 for 15 min at RT, followed by staining with 1 μM DAPI (Sigma-Aldrich). Fluorescent images were obtained with a confocal laser scanning microscope (Leica Microsystems, Heidelberg GmbH, Mannheim, Germany).

### 2.8. Statistical Analysis

The data were analyzed using a one-way analysis of variance (ANOVA) followed by the Tukey-Kramer test. All analyses were performed using the GraphPad Prism (version 6.03) software. Significant differences between the experimental groups were established as *p*-values less than 0.001(***), 0.01(**), or 0.05(*).

## 3. Results

### 3.1. FPV Infection Fails to Activate IFN-β and Interrupts SeV-Mediated IFN-β Induction

**Figure 1:**
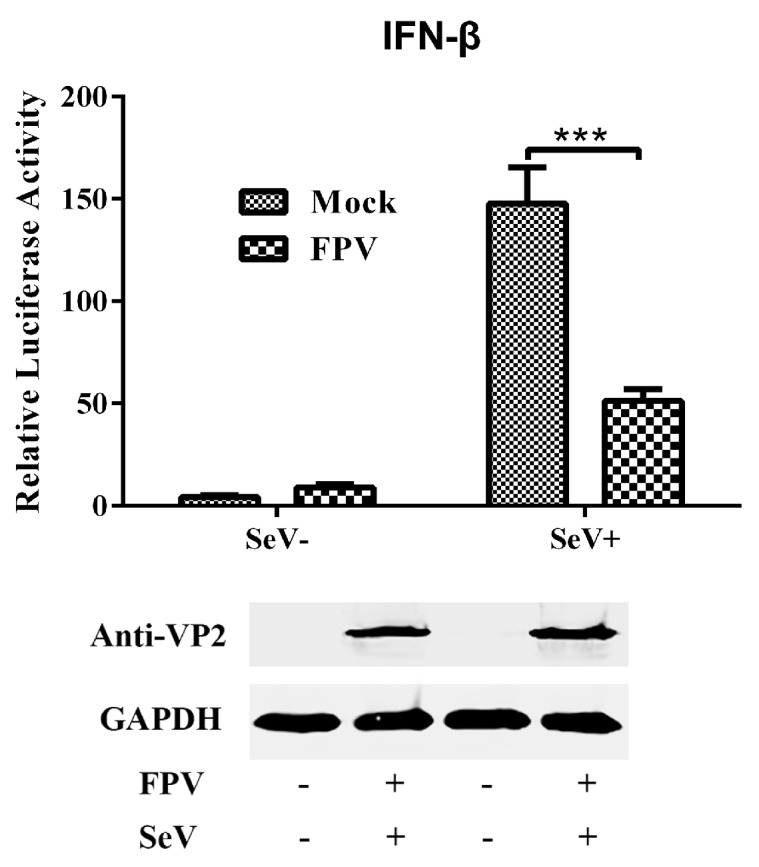
Effect of feline panleucopenia virus (FPV) on interferon (IFN)-β production and Sendai virus (SeV)-mediated IFN-β production as indicated by luciferase activity in F81 cells. The cells in this experiment were co-transfected with IFN-β-Luc and the Renilla luciferase construct pRL-TK, followed by a mock infection or infection with FPV. After 12 h of co-transfection, the cells were stimulated with SeV (SeV+) or left untreated (SeV-). The data represent the relative firefly luciferase activity normalized to the Renilla luciferase activity. The data represent the mean values of three independent experiments. The error bars represent standard deviations, and the asterisks indicate significant differences (*: *p* < 0.05; **: *p* < 0.01; ***: *p* < 0.001) between groups. The FPV infection was monitored by immunoblotting using a mouse anti-capsid protein 2 (VP2) antibody, and glyceraldehyde 3-phosphate dehydrogenase (GAPDH) was used as a loading control.

To explore if FPV affects IFN-β induction, the IFN-β promoter luciferase reporter system was used to analyze IFN-β expression after FPV infection. F81 cells were co-transfected with the luciferase reporter plasmid IFN-β-Luc and the internal control plasmid pRL-TK, followed by a mock infection or infection with FPV. After 12 h of co-transfection, the cells were stimulated with SeV (SeV+) or were left untreated (SeV-). The cells were lysed 8–12 h after stimulation, and both the firefly and Renilla luciferase activities were evaluated. As shown in Figure 1, without SeV stimulation, no significant differences in luciferase activity were detected between cells infected with FPV and mock-infected cells, suggesting that FPV infection could not activate the IFN-β promoter. Next, with SeV stimulation, the cells infected with FPV produced a significantly lower level of luciferase activity compared with the mock-infected cells, which indicated that FPV infection inhibits SeV-induced IFN-β promoter activity and interrupts SeV-mediated IFN-β production.

### 3.2. FPV NS2 as a Negative Regulator Impedes SeV-Mediated IFN-β Induction

**Figure 2:**
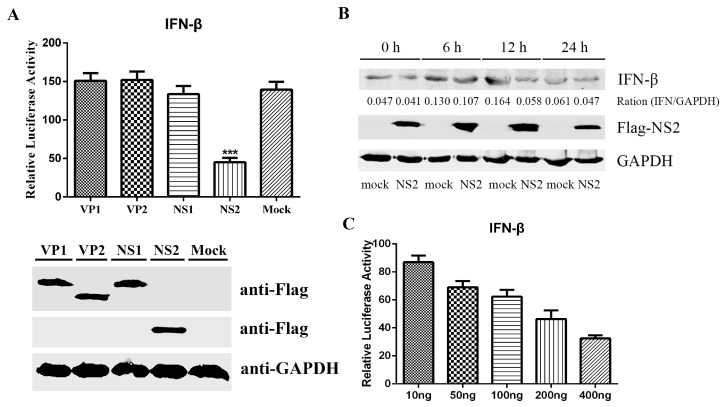
FPV NS2 as a negative regulator impedes SeV-mediated IFN-β induction. (**A**) Effects of protein-coding genes of FPV on the SeV-induced IFN-β promoter activation in F81 cells. The cells in this experiment were co-transfected with IFN-β-Luc, the Renilla luciferase construct pRL-TK and one of the recombinant plasmids pFlag-vp1, pFlag-vp2, pFlag-ns1, or pFlag-ns2. Twenty-four hours later, the cells were stimulated with SeV. The luciferase activity was measured at 12 h after simulation. The values were normalized to the Renilla activity. The data represent the mean values of three independent experiments. The error bars represent standard deviations, and “*” indicates significant differences (*p* < 0.05) between groups. The expression of VP1, VP2, NS1, or NS2 was monitored by immunoblotting using a mouse anti-Flag antibody; GAPDH was used as a loading control. (**B**) The SeV-mediated IFN-β expression is disrupted by NS2. F81 cells were transfected with p3×Flag-NS2. At 12 h post transfection, the cells were inoculated with SeV. The cell lysates at 0, 6, 12 and 24 h after SeV infection were analyzed by immunoblotting (IB) using anti-Flag and anti-IFN-β antibodies. (**C**). NS2 inhibits IFN promoter activity in a dose-dependent manner. F81 cells were co-transfected with IFN-β-Luc, pRL-TK and different amounts of p3×Flag-NS2 (10, 50, 100, 200 or 400 ng). At 12 h post transfection, the cells were inoculated with SeV. Twelve hours after infection, the cells were harvested, and the luciferase activities were measured. The values were normalized to the Renilla activity. The data represent the mean values of three independent experiments. The error bars represent standard deviations, and the asterisks indicate significant differences (*: *p* < 0.05; **: *p* < 0.01; ***: *p* < 0.001) between groups.

We next evaluated which viral protein(s) could modulate the IFN-β induction. F81 cells were co-transfected with IFN-β-Luc, pRL-TK, and a plasmid expressing one of the FPV viral proteins (VP1, VP2, NS1, and NS2). As shown in Figure 2A, we found that NS2 could significantly inhibit the activation of the IFN-β promoter. We then validated the NS2-mediated inhibition of the IFN-β induction by measuring the IFN-β expression in cells stimulated with SeV after transfection with NS2 (Figure 2B). In accordance with the IFN-β promoter activity, in the presence of NS2, the expression of IFN-β was decreased after the cells were infected with SeV for 12 h compared with the mock group without NS2 transfection. These results indicated that FPV NS2 is a negative regulator of type I IFN induction and that the inhibitory effect occurred in a dose-dependent manner (Figure 2C). These results consistently supported the suppression of IFN-β induction by NS2.

### 3.3. NS2 Interrupts the SeV-Mediated Activation of IFN-β by Blocking the IRF3 Pathway

**Figure 3:**
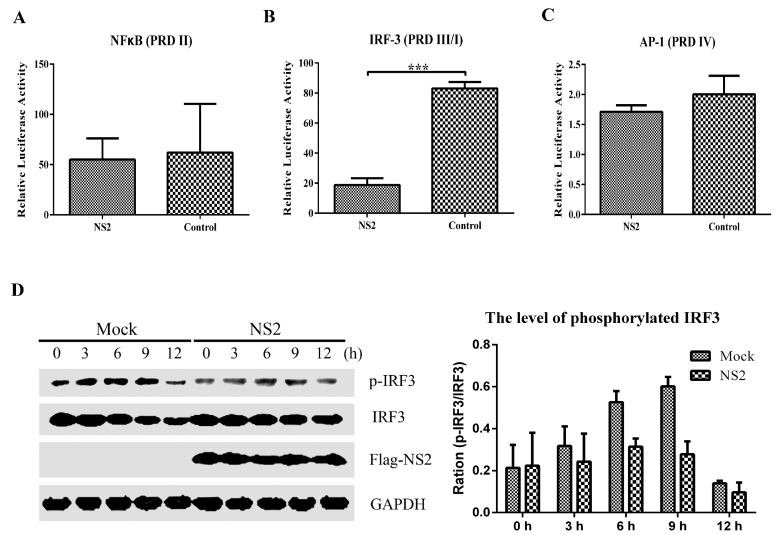
Inhibition of the activity of transcription factors on specific PRDs of the IFN-β promoter by NS2. F81 cells were co-transfected with p3×Flag-NS2, pRL-TK and either p3×PRDII-Luc (**A**), PRDIII/I-Luc (**B**), or p6×PRDIV-Luc (**C**). At 12 h post-transfection, the cells were inoculated with SeV. Twelve hours after infection, the cells were harvested and the luciferase activities were measured. The values were normalized to the Renilla activity. The error bars represent standard deviations, and the asterisks indicate significant differences (*: *p* < 0.05; **: *p* < 0.01; ***: *p* < 0.001) between groups. (**D**) The phosphorylation levels of IRF3 in the cells stimulated with SeV after transfection with NS2. F81 cells were transfected with p3×Flag-NS2. At 12 h post-transfection, the cells were inoculated with SeV. The cell lysates at 0, 3, 6, 9 and 12 h after SeV infection were analyzed by immunoblotting using antibodies against IRF3 or phosphorylated IRF3 and the quantification of relative p-IRF3 band intensities to IRF3 was showed with histogram.

To investigate which signal pathway(s) of type I IFN induction were inhibited by NS2, F81 cells were co-transfected with the luciferase reporter plasmids pNF-κB-Luc, pIRF3-Luc or pAP-1-Luc, pRL-TK, and p3×Flag-NS2 (p3×Flag as control). After 12 h of co-transfection, the cells were stimulated with SeV. The cells were lysed 12 h after infection, and the cell supernatant was used to evaluate the firefly and Renilla luciferase activities. Compared to the cells transfected with pIRF3-Luc and empty vector, the luciferase activity of the cells transfected with pIRF3-Luc and p3×Flag-NS2 were significantly reduced, as shown in Figure 3B. However, the same results were not observed in cells transfected with pNF-κB-Luc and p3×Flag-NS2 or pAP-1-Luc and p3×Flag-NS2 (Figure 3A,C). Furthermore, we examined the phosphorylation level of IRF3 in the cells stimulated with SeV after transfection with NS2, as shown in Figure 3D. From 0 h to 9 h, the level of phosphorylated IRF3 increased after SeV inoculation, but in the presence of NS2, the level of phosphorylated IRF3 decreased after SeV infection. These results indicated that NS2 could impede SeV-mediated activation of the transcription factor IRF3 but does not suppress the activation of the transcription factors NF-κB and AP-1. Hence, we hypothesized that NS2 interrupts the SeV-mediated activation of IFN-β by blocking the IRF3 pathway.

**Figure 4:**
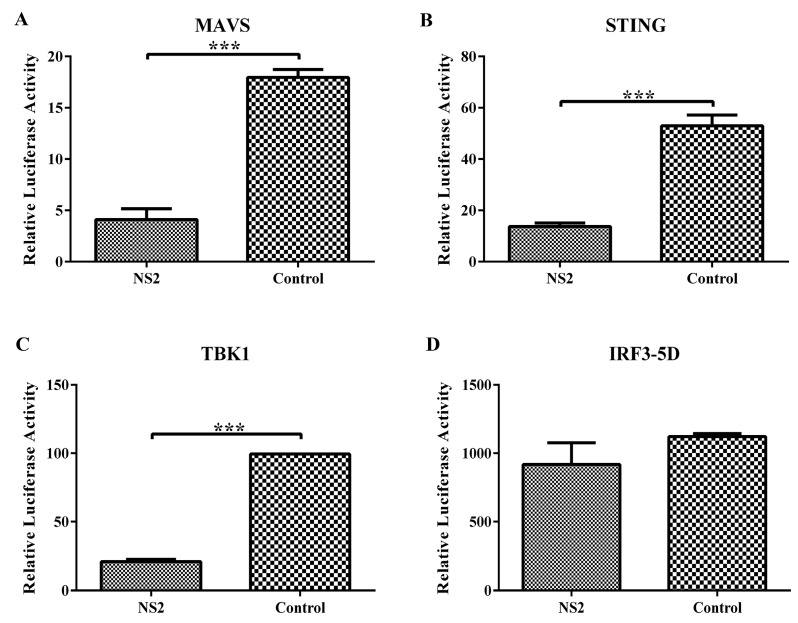
FPV NS2 blocks interferon induction at a step upstream of IRF3. F81 cells were co-transfected with IRF3-Luc, pRL-TK, p3×Flag-NS2 and a plasmid expressing one of the molecules in the IRF3 signaling pathway: MAVS (**A**), STING (**B**), TBK1 (**C**), and IRF3-5D (**D**), respectively. The cell lysates were used to measure the firefly and Renilla luciferase activities at 24 h post-transfection. The values were normalized to the Renilla activity. The data represent the mean values of three independent experiments. The error bars represent standard deviations, and the asterisks indicate significant differences (*: *p* < 0.05; **: *p* < 0.01; ***: *p* < 0.001) between groups.

Next, we screened which adaptor molecules in the IRF3 pathway were inhibited by NS2. F81 cells were co-transfected with IRF3-Luc, p3×Flag-NS2 and a plasmid expressing one of several adaptors in the IRF3 signaling pathway, including MAVS, STING, TBK1, and IRF3-5D. The cell lysates were used to measure the firefly and Renilla luciferase activities at 24 h post-transfection. As shown in Figure 4, the transfection of FPV NS2 significantly suppressed the activation of the IFN-β promoter stimulated by molecules upstream of IRF3 such as MAVS, STING, and TBK1 but did not counteract the IRF3-5D-mediated IRF3 activation. These results provide evidence supporting the hypothesis that FPV NS2 interrupts SeV-mediated IFN-β induction by acting on the proteins upstream of IRF3 and that TBK1 appears to be a target protein of NS2 suppression.

### 3.4. NS2 Can Interact Directly with TBK1 and Disrupt the TBK1–STING Interaction

**Figure 5:**
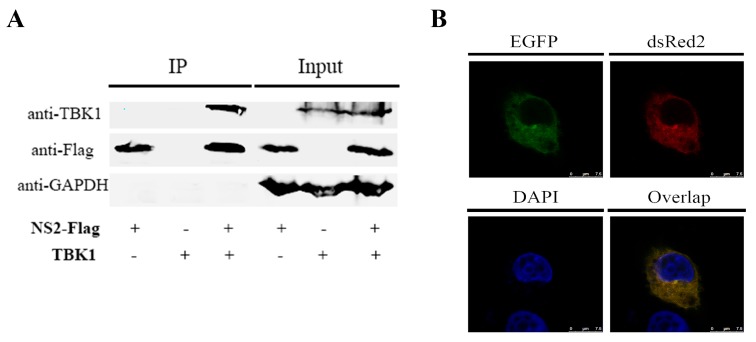
Analysis of the interaction between NS2 and TBK1. (**A**) Flag-tagged NS2 was co-transfected with Myc-tagged TBK1 into F81 cells for 36 h. The cell lysates were immunoprecipitated using ANTI-FLAG M2 magnetic beads. The whole-cell lysates and immunoprecipitation complexes were analyzed by immunoblotting using anti-Flag or anti-Myc antibodies. (**B**) F81 cells were co-transfected with the pDsred-TBK1 plasmid and pEGFP-NS2 plasmid. Twenty-four hours after transfection, the nuclei were stained with DAPI. The fluorescent images of the cells were obtained with a confocal laser scanning microscope.

To confirm whether FPV NS2 interacted with TBK1, co-immunoprecipitation and western blotting assays were used, and cells were co-transfected with p3×Flag-NS2 and pMyc-TBK1. As shown in Figure 5A, Flag-tagged NS2 interacted with Myc-tagged TBK1, demonstrating the association between NS2 and TBK1. Furthermore, this result was validated by the confocal assay. The results showed the co-localization of NS2 and TBK1 in the cytoplasm, with a co-localization coefficient of 0.934 based on the digital analysis of cell images. Taken together, our data demonstrate that TBK1 interacts directly with FPV NS2.

**Figure 6:**
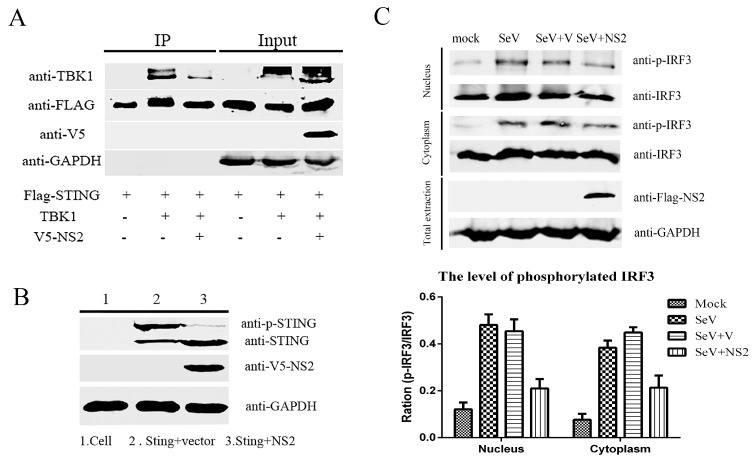
NS2 disrupts the TBK1–STING interaction and reduces the phosphorylation of STING and downstream IRF3. (**A**) F81 cells were transfected with Flag-tagged STING, Myc-tagged TBK1 and V5-tagged NS2 for 36 h. The cell lysates were subjected to immunoprecipitation with ANTI-FLAG M2 magnetic beads. The whole-cell lysates and IP complexes were analyzed by western blot using anti-Flag or anti-Myc antibodies. (**B**) F81 cells were transfected with Flag-tagged STING and V5-tagged NS2 for 36 h. The cell lysates were resolved by SDS-PAGE, after which they were analyzed by western blot with antibodies against STING or phosphorylated STING. (**C**) F81 cells were transfected with Flag-tagged NS2. Twenty-four hours later, the cells were stimulated with SeV. The cytoplasmic and nuclear extracts of the F81 cells were separated at 6 h after simulation and analyzed by western blot with antibodies against IRF3 or phosphorylated IRF3 and the quantification of relative p-IRF3 band intensities to IRF3 was showed with histogram.

To determine whether the interaction between NS2 and TBK1 affects the function of TBK1, we assessed the impact of NS2 on the assembly of the STING-TBK1 complex. p3×Flag-STING and pMyc-TBK1 were co-transfected into F81 cells in the absence or presence of pV5-NS2. The cell lysates were then co-immunoprecipitated. We observed that the co-immunoprecipitation of STING with TBK1 was disrupted in the presence of NS2. Moreover, the phosphorylation of STING was also reduced when the cells were co-transfected with p3×Flag-STING and pV5-NS2, as determined by western blot analysis (Figure 6). Furthermore, the phosphorylation of IRF3 in the cytoplasm and nucleus was also attenuated in the presence of NS2. These results indicated that the interaction between NS2 and TBK1 inhibited the recruitment of TBK1 to STING, which reduced the phosphorylation of STING and the phosphorylation of downstream IRF3, leading to the inhibition of the IRF3 signaling pathway. Thus, NS2 inhibits the function of the IRF3 signaling pathway by interacting with TBK1 to disrupt the TBK1–STING interaction.

### 3.5. The C-terminus Plus the Coiled Coil Domain of NS2 can Inhibit the IRF3 Signaling Pathway to the Same Extent as Full-Length NS2

**Figure 7:**
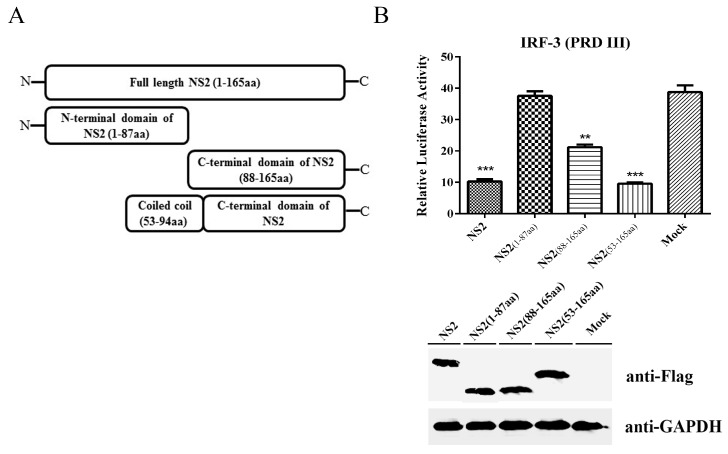
The C-terminus plus the coiled coil domain of NS2 can inhibit the IRF3 signaling pathway. (**A**) Schematic diagram of the truncated NS2 constructs. (**B**) F81 cells were co-transfected with IRF3-Luc, pRL-TK and plasmids expressing either the intact NS2, the N-terminal domain of NS2, the C-terminal domain of NS2 or the C-terminal domain plus the coiled coil domain of NS2. The effect of each truncated NS2 on the expression of the luciferase gene under the control of the IRF3 binding region PRDIII/I of the feline IFN-β promoter was determined using a luciferase assay. The values were normalized to the Renilla activity. The data represent the mean values of three independent experiments. The error bars represent standard deviations, and the asterisks indicate significant differences (*: *p* < 0.05; **: *p* < 0.01; ***: *p* < 0.001) between groups. The expression of the intact NS2, the N-terminal domain of NS2, the C-terminal domain of NS2 or the C-terminal domain plus the coiled coil domain of NS2 was monitored by immunoblotting using a mouse anti-Flag antibody; GAPDH was used as a loading control.

To define the regulatory elements within NS2, we analyzed the sequence and structure of the NS2 protein and created truncated forms NS2, including the N-terminal domain (1–87aa), which is the same sequence as the NS1 N-terminal domain, a C-terminal domain (88–165aa) and the C-terminal domain plus the coiled coil domain (53–165aa). The effect of each truncated form of NS2 on the expression of the luciferase gene under the control of the IRF3 binding region PRDIII/I of the feline IFN-β promoter was determined using a luciferase assay. As shown in Figure 7, the N-terminal domain could not affect the activity of the feline IFN-β promoter through the PRDIII/I region. Compared with the full-length NS2, the C-terminal domain of NS2 exhibited reduced ability to inhibit the reporter gene expression, with about 50% of inhibitory ability of the full-length NS2. Interestingly and importantly, the construct containing the C-terminus plus the coiled coil domain of NS2 produced an inhibitory effect similar to that of the full-length NS2. These findings suggest that the C-terminus plus the coiled coil domain of NS2 are the functional determinants of the NS2-mediated inhibition of the IRF3 signaling pathway.

## 4. Discussion

Viruses of the carnivore parvovirus group infect a wide range of hosts and cause severe diseases. However, the mechanism of their evasion of the innate immune system has rarely been reported. In the *Parvoviridae* family, only a few viruses, such as minute virus of mice (MVM), porcine parvovirus (PPV), and porcine bocavirus (PBoV), have been reported to block IFN-β production to attenuate innate immune responses [12,13,14]. In the present study, we focused on the immune evasion of FPV and showed the antagonistic function of the FPV NS2 protein against the IRF3 signaling pathway to inhibit IFN-β induction.

To combat the host antiviral effects, viruses have evolved elaborate mechanisms to antagonize the innate immune response [15,16]. The inhibition of interferon transcription is a common method viruses use to escape the innate immune response [3]. In the present study, we first found that infection with the FPV strain Philips-Roxane failed to activate IFN-β transcription but antagonized the type I IFN response stimulated by SeV in F81 cells. Next, we overexpressed the structural and nonstructural proteins of FPV and found that NS2 could inhibit the IFN-β induction. In this experiment，the expression of luciferase gene induced by IFN-β promoter can be accumulated, but the induction of IFN-α/β during the early phase of viral infection is detectable, then the negative feedback pathway against IFN-α/β is activated and begins to degrade the IFN-α/β. So, the different expression of IFN-β was significant only at some time points, but the significantly different expression of luciferase gene presented during the whole infection stage, which contributed that the suppression result in luciferase reporter assays were more significant than that in western blot. The initiation of IFN expression is tightly regulated by transcription factors consisting of IRFs, NF-κB, and AP-1. These transcription factors bind specific PRD motifs in the IFN-β promoter, and an association with the transcriptional coactivator CREB-binding protein (CBP) and p300 leads to the initiation of IFN transcription in the nucleus. In the current study, transfection with NS2 only inhibited the luciferase activity of the PRDIII motif, which has specific affinity to IRF3, and did not interrupt the luciferase activity of PRDII, which has specific affinity to NF-κB and PRD IV, which has specific affinity to AP-1. These results indicated that NS2 impedes the IRF3 signaling pathway to interrupt the SeV-mediated activation of IFN production.

To pinpoint the key step and the target protein of the NS2-mediated suppression of IFN-β induction, effectors in the IRF3 signaling pathway were investigated by epistatic analysis. The induction of type I IFN production is governed by signal transduction pathways in which an activation signal is relayed by a cascade of signaling proteins to IRF3 transcription factors which then turn on IFN promoters [17]. Theoretically, NS2 should suppress the effect of all upstream inducers and have no influence on the effect of its downstream effectors. Therefore, the action point of NS2 can be determined by assessing its suppressive effects on a series of transducer proteins. In this study, our findings demonstrate that FPV NS2 inhibited MAVS-, STING-, and TBK1-directed IFN-transcription (>80%) but failed to inhibit IFN-induction directed by constitutively active IRF3-5D. We therefore reasoned that NS2 might act at the TBK1 step. As a phosphokinase, TBK1 plays critical roles in the IRF3 signaling pathway. TBK1 and IRF3 can be recruited by STING to form a complex that facilitates TBK1 phosphorylation and activation followed by STING and IRF3 phosphorylation by TBK1, thus leading to the activation of the IFN-β response and triggering the host immune response [18,19]. In the present study, we found that NS2 could interact with TBK1 and significantly inhibit both the binding of TBK1 to STING and the subsequent phosphorylation of STING and IRF3. These results indicate that NS2 inhibits the function of the IRF3 signaling pathway by preventing TBK1 from binding to STING. Thus, we report that NS2 can block the interaction between TBK1 and STING to disturb the TBK1-mediated IRF3 phosphorylation, which is the key step in the NS2-mediated suppression of IFN-β induction.

The functions of NS2 in the viral life cycle or replication are not well understood [20]. FPV NS2 is formed from a spliced transcribed message, with its 87 N-terminal amino acids, which it shares with nonstructural protein 1 (NS1), being joined to 78 amino acids from an alternative open reading frame, and has been shown to have little effect on efficient viral DNA replication and the assembly of viral capsid proteins. In the current study, we demonstrated that FPV NS2 could significantly suppress IFN expression. To define the important regulatory elements within NS2, the effects of several truncated forms of NS2 on the inhibition of SeV-induced IFN-β induction were examined. In accordance with the function of NS1 in anti-IFN activity, the N-terminal domain of NS2 could not inhibit reporter gene expression. However, the C-terminal domain of NS2 had 50% of the inhibitory ability of full-length NS2. By analyzing the sequence and structure of NS2, we found a coiled coil domain between the N-terminal domain and C-terminal domain. Coiled coil-mediated protein-protein interactions have been frequently detected [21]. A truncated form of NS2 in which the coiled coil domain was joined to the C-terminal domain produced a similar inhibitory effect as full-length NS2. These results indicated that the C-terminus plus the coiled coil domain of NS2 are the key regulatory elements required for FPV to suppress type I IFN induction. Although the specific mechanism needs further investigation, due to the high conservation of the NS2 sequence, the function of the NS2 protein in inhibiting host innate immunity may be widespread in the carnivore parvovirus subgroup.

In summary, this study is the first report identifying the viral proteins encoded by FPV that are responsible for inhibiting the induction of IFN-β. We found that NS2 inhibits the induction of IFN-β by targeting TBK1 to prevent it from interacting with STING, thereby inhibiting the downstream IRF3 phosphorylation and resulting in an inhibition of the IRF3 signaling pathway. The present study has shed light on a novel function of the FPV NS2 protein as a negative regulator of IFN-β production. Moreover, these findings contribute to our understanding of the molecular mechanisms of innate immunity evasion strategies utilized by FPV and the outcomes of infection such as the establishment of a persistent FPV infection.

## Supplementary Materials

The following are available online at www.mdpi.com/1999-4915/9/1/23/s1, Table S1: Primers used in the study.
