# Supplementary material for: Feline Panleucopenia Virus NS2 Suppresses the Host IFN-β Induction by Disrupting the Interaction between TBK1 and STING
Source: Viruses. 2017 Jan 23;9(1):23. doi: 10.3390/v9010023 (PMC5294992; doi:10.3390/v9010023)
Supplement: Supplementary file 1 [file viruses-09-00023-s001.pdf]

Supplementary Materials: Feline Panleucopenia Virus NS2 Suppresses the Host IFN-β Induction by Disrupting the Interaction between TBK1 and STING

Hongtao Kang [1†], Dafei Liu [1†], Jin Tian [1], Xiaoliang Hu [1], Xiaozhan Zhang [1], Hang Yin [2], Hongxia Wu [1], Chunguo Liu [1], Dongchun Guo [1], Zhijie Li [1], Qian Jiang [1], Jiasen Liu [1], and Liandong Qu [1,*]

Table S1. Primers used in the study.

Primer	Sequence 5′- 3′	Usage
F-NS1F	GGGGTACCAATGTCTGGCAACCAGTATACTG	Flag-NS1, NS2
F-NS1R	CGGGATCCTTAATCCAAGTCGTCTCGAAAATC	Flag-NS1
F-NS2R	CGGGATCCTCAGAAGTAAAGATGGCTCTC	Flag-NS2
F-VP1F	GGGGTACCAATGGCACCTCCGGCAAAGAG	Flag-VP1
F-VP1R	CGGGATCCTTAATATAATTTTCTAGGTGCTAG	Flag-VP1, VP2
F-VP2F	GGGGTACCAATGAGTGATGGAGCAGTTCAAC	Flag-VP2
XBTBK1F	GGGCTCGAGCTATGCAGAGCACTTCTAATCAT	DsRed2-TBK1
XBTBK1R	AAA GGATCCCTAAAGACAGTCAACGTTGCG	DsRed2-TBK1
XBNS2F	GGGG CTCGAGCTATGTCTGGCAACCAGTATA	EGFP-NS2
XBNS2R	GGG GGATCCTCAGAAGTAAAGATGGCTCTCA	EGFP-NS2
BXNS2F	GGGG GGATCCATGTCTGGCAACCAGTATA	NS2-V5
BXNS2R	GGG CTCGAGCGGAAGTAAAGATGGCTCTCA	NS2-V5
NS2-1F	GG GGTACCAATGTCTGGCAACCAGTATACTG	1-87aa NS2
NS2-261R	GGGG GGATCCTCACTTTTTGGCGAGACTATC	1-87aa NS2
NS2-262F	GG GGTACCAATGGTTGATAGTCTCGCCAAAAAG	88-165aa NS2
NS2-498R	GGGG GGATCCTCAGAAGTAAAGATGG	88, 53-165aa NS2
NS2-157F	GGGG GGTACCAATGCCAATTCAAAATGAAGAGCT	53-165aa NS2

© 2017 by the authors. Submitted for possible open access publication under the terms and conditions of the Creative Commons Attribution (CC BY) license (http://creativecommons.org/licenses/by/4.0/).
